# Retinoma: spontaneous regression of retinoblastoma or benign manifestation of the mutation?

**DOI:** 10.1038/bjc.1982.87

**Published:** 1982-04

**Authors:** B. L. Gallie, R. M. Ellsworth, D. H. Abramson, R. A. Phillips

## Abstract

**Images:**


Br. J. Cancer (1982) 45, 513

### RETINOMA: SPONTANEOUS REGRESSION OF RETINOBLASTOMA OR BENIGN MANIFESTATION OF THE MUTATION?

B. L. GALLIE*, R. M. ELLSWORTH†‡, D. H. ABRAMSON†‡, AND R. A. PHILLIPS*

*From the *Ontario Cancer Institute, Toronto Canada, and the †Harkness Eye Institute, Columbia University, New York, USA*

Received 13 May 1981 Accepted 23 November 1981

**Summary.—Non-progressive retinal lesions, observed in patients known to carry the gene for retinoblastoma, have in the past been called "spontaneous regression" of retinoblastoma. This term suggests shrinkage of a malignant growth, perhaps in response to some host defence mechanism. On the basis of observations on 30 patients, we propose that the term "retinoma" would be less presumptive and more suitable. Retinoma is clinically defined as a translucent, grey, elevated mass extending into the vitreous from the retina, frequently associated with calcified foci and pigment-epithelium hyperplasia. The diagnosis of retinoma strongly suggests the presence of the retinoblastoma gene, necessitating genetic counselling and frequent observation of the retinas in the individual and his offspring. We suggest that the same mutations can cause either retinoma or retinoblastoma: benign hyperplastic nodules or retinoma when the mutations occur in relatively mature retinoblasts; and malignant retinoblastoma when the same mutations arise in immature retinoblasts.**

RETINOBLASTOMA is a rare malignant tumour of embryonic retinal cells, associated with a dominantly inherited autosomal gene in one-third of cases. Although retinoblastoma is generally considered fatal without treatment, the apparent frequency of spontaneous regression may be as high as 1% (Gallie *et al.*, 1977*a*). In contrast, Cole (1974) reviewed the evidence for spontaneous regression in all other malignant diseases and concluded that the overall frequency was 1/80,000 or about 1000 times less frequent than in retinoblastoma.

Two distinct clinical entities have been labelled "spontaneous regression of retinoblastoma". The first entity, of shrunken, scarred, calcified eyes (phthisis bulbi) probably results from complete intraocular ischaemic necrosis of the tumour (Andersen & Jensen, 1974). The mechanisms leading to the second entity, non-progressive retinal lesions in otherwise functional eyes, are less obvious. To obtain a better understanding of the biological and possible therapeutic significance of the non-progressive retinal lesions, we searched through the records of 1500 retinoblastoma patients and their relatives seen at the Harkness Eye Institute between 1961 and 1979, and identified 28 individuals who had been diagnosed as having this form of spontaneous regression of retinoblastoma; 2 more patients were seen at the Toronto clinic. Consideration of this clinical material leads us to propose the new term "retinoma" to designate these distinctive retinal lesions, highly associated with retinoblastoma but lacking malignant characteristics.

### METHODS

All the individuals considered possibly to manifest spontaneous regression of retino-

Correspondence and reprints requests to Dr Brenda L. Gallie, Jennison Surgical Research Laboratory, Wellesley Hospital, 160 Wellesley Street East, Toronto, Ontario, Canada M4Y 1J3

‡ Present address: The New York Hospital, Cornell University Medical Center, 525 East 68th Street, New York, New York 10021

blastoma had at least one characteristic lesion of the retina, observed through a dilated pupil with an indirect ophthalmoscope. These lesions did not change on repeated examination. In addition, all patients were free of actively growing retinoblastoma and had received no radiotherapy or chemotherapy. A detailed family history was obtained from all individuals to determine whether or not any relatives had had retinoblastoma.

### RESULTS

Non-progressive retinal lesions, defined by the 3 features shown in Fig. 1, were present in 34 eyes of 30 patients, 15 male and 15 female. Compared to the cases of retinoblastoma seen at the Harkness Eye Institute, a frequency of 1·8% (28/1500) can be calculated, but the referral pattern of this clinic makes this estimate inaccurate. A homogeneous, translucent, grey, elevated mass resembling "fish flesh" extended from the retinal surface into the vitreous cavity in 90% of the lesions, with retinal blood vessels irregularly deviating into the masses. Opaque, white nodules, apparently containing calcium, and having the appearance of "cottage cheese" occurred in 75% of the lesions. Retinal pigment-epithelium migration and proliferation in areas underlying and adjacent to the nodules caused irregular pigment distribution around 61% of the lesions. Two or more of these features in each patient permitted inclusion in ths study, but some individual lesions displayed only one feature. Fluorescein angiography (Fig. 2) shows that the lesions are supplied mainly by the retinal circulation. With repeated observation over intervals of 1–28 years (mean 10) the lesions failed to change. For the purposes of this paper, we will use the term "retinoma" to refer to these essen-

FIG. 1.—The 3 features of retinoma are demonstrated in the fundus photograph of Case 5: homogeneous, translucent, grey mass extending into vitreous (A), "cottage cheese" calcification (B), and proliferation and migration of the pigment epithelium (C).

FIG. 2.—Fluorescein angiography on the retinoma of Case 395, revealing retinal blood vessels supplying retinoma (↑) and choroid circulation visible through the pigment-epithelium defect (↑↑).

tially benign retinal tumours with clinicopathological features suggesting retinoblastoma, in order to avoid the presumption that they are indeed retinoblastoma.

The Table summarizes the clinical and genetic data for the 30 cases, subdivided into 5 groups. Individuals in Group A (7 cases) had a single retinoma in one eye with no past or family history of retinoblastoma. Individuals in Group B (3) had multiple, bilateral retinomas, with no family history and no previous pathological evidence of retinoblastoma. The retinomas in Groups A and B were detected on routine eye examination. The mean age at diagnosis of retinomas was 8 years (range 5–16).

All retinoma cases had normal intelligence and showed no malformations suggesting deletion of chromosome 13. Case 31 has a familial translocation 13 : 14, but this does not segregate with retinoblastoma in his daughter and is probably coincidental.

Individuals in Group C (6) had single or multiple retinomas and a family history of retinoblastoma, but the individuals themselves were never diagnosed or treated for retinoblastoma. Fatal progression of retinoblastoma occurred in relatives of 3 individuals in this group: the daughter of Case 1, a sibling of Case 36, and the daughter of Case 115. The retinomas in Case 31 were discovered on routine examination at the age of 24 years, and he was originally placed in Group B. Recently, his newborn daughter was found to have retinoblastoma and he was moved to Group C. The other individuals in this group were identified when relatives were found to have retinoblastoma. Their mean age at diagnosis of retinoma was 18 years (range 3–29).

Patients in Groups D and E had uni-

TABLE.—*Summary of clinical data*

| Group | Case no. | Sex | Ocular lesions* (r = retinoma) | Age at detection (yrs) | Resultant visual acuity | Children with retinoblastoma/ total children | Other relatives with retinoblastoma |
|---|---|---|---|---|---|---|---|
| A | 5 | M | OD: r (abc) | 5 | 20/20 | 0/0 | None |
| | 62 | F | OS: r (ac) | 7 | 20/20 | 0/0 | None |
| | 120 | M | OD: r (abc) | 9 | 20/20 | 0/0 | None |
| | 139 | F | OS: r (abc) | 10 | 20/20 | 0/0 | Unknown/adopted |
| | 154 | M | OS: r (abc) | 7 | 20/20 | 0/0 | None |
| | 221 | F | OS: r (ab) | 16 | 20/20 | 0/0 | None |
| | 233 | F† | OD: r (abc) | 6 | 20/20 | 0/0 | None |
| B | 57 | M | OD: 4r (b, ab, a, a) | 8 | 20/20 | 0/0 | None |
| | | | OS: r (ab) | | 20/20 | | |
| | 266 | M | OD: 3r (ab, a, a) | 7 | 20/20 | 0/0 | None |
| | 237 | M | OD: 2r (ab, ac) | 9 | 20/20 | | |
| | | | OS: cataract; posterior calcified mass by ultrasound | | NLP‡ | 0/0 | None |
| C | 1 | F | OD: 2r (abc, ab) | 29 | 20/20 | 1/1 | None |
| | 31 | M† | OD: 2r (abc, abc) | 24 | 20/20 | 1/1 | None |
| | | | OS: 2r (abc, abc) | | 20/80 | | |
| | 36 | F | Bilateral r (ab, abc) | 24 | 20/20 | 3/5 | 1 sibling |
| | 115 | F | Bilateral r (abc, abc) | 22 | 20/20; 20/60 | 4/5 | None |
| | 128 | M† | OD: 2r (ac, a) | 5 | 20/70 | 0/0 | Father, 2 siblings |
| | 346 | M | OD: r (abc) | 3 | 20/200 | 0/0 | 1 sibling |
| D | 89 | M | OD: en (RB)§ | 8 | — | 0/0 | None |
| | | | OS: r (abc) | 13 | 20/20 | | |
| | 200 | M | OD: en (phthisis bulbi) | 32 | — | 0/0 | None |
| | | | OS: r (ac) | 32 | 20/400 | | |
| | 205 | F | OD: r (abc) | 4 | 20/20 | 0/0 | None |
| | | | OS: en (RB) | 1 2/12 | — | | |
| | 396 | F | OD: 2r (a, b) | 40 | 20/25 | 0/2 | None |
| | | | OS: en (RB) | 2 | — | | |
| E | 28 | F | OD: en (RB) | 3/12 | — | 2/2 | None |
| | | | OS: 2r (abc, ac) | 26 | 20/20 | | |
| | 70 | F | OD: en (RB) | 2 | — | 1/1 | Father, 1 sibling |
| | | | OS: 2r (abc, ac) | 18 | 20/20 | | |
| | 159 | F | OD: 2r (ab, a) | 18 | 20/20 | 1/2 | None |
| | | | OS: en (RB) | 6/12 | — | | |
| | 165 | M | OD: en (RB) | 9/12 | — | 2/2 | Granddaughter |
| | | | OS: 2r (ab, ac) glaucoma | 35 | 20/20 | | |
| | 215 | M | OD: r (abc) | 25 | 20/50 | 2/2 | None |
| | | | OS: en (RB) | 6/12 | — | | |
| | 232 | M | OD: en (RB) | 6/12 | — | 1/4 | None |
| | | | OS: r (abc) | 30 | 20/40 | | |
| | 283 | F | OD: en (RB) | 2 | — | 0/0 | 1 sibling, grandmother |
| | | | OS: r (ac) | 28 | 20/20 | | |
| | 304 | F | OD: r (ac) | 24 | 20/30 | 1/1 | None |
| | | | OS: en (RB) | 2 | — | | |
| | 306 | M | OD: en (RB) | 2 | — | 2/2 | Unknown |
| | | | OS: r (bc) | 25 | Unknown | | |
| | 395 | F | OD: r (abc) | 40 | 20/25 | 1/2 | None |
| | | | OS: en (phthisis bulbi) | 8 | — | | |

* The letters indicate the properties of the retinoma; each retinoma may have 1, 2, or 3 of the following attributes: (a) grey translucent mass; (b) "cottage cheese" calcification; (c) changes in the surrounding pigment epithelium. OS, OD. Left and right eyes, respectively. en = enucleated.

† Previously reported: Case 233 in Rubin & Kaufman (1969); Case 31 in Morris & Lapiana (1974); and Case 128 in Brockhurst & Donaldson (1970).

‡ No light perception.

§ RB: pathological confirmation of retinoblastoma.

FIG. 3.—Pedigrees of 2 families from the Table. Family I: Case 115 with 2 husbands, had 4 children with retinoblastoma and 1 normal child, before characteristic lesions were found in her eyes. Family II: Cases 70 and 165 each had 1 eye enucleated with retinoblastoma in childhood, and retinomas were found in the remaining eyes when retinoblastoma was detected in their offspring.

lateral tumours treated by enucleation with pathological confirmation of the diagnosis of retinoblastoma. With the exception of Case 200 (see below), enucleation was performed in early childhood (mean age 1·7 years, range 3 months to 8 years). Subsequently, at a mean age of 25 years (range 4–40), these individuals were found on examination of their remaining eye to have small retinomas. Patients in Group D (4) had no family history of retinoblastoma but those in Group E (10) had affected family members.

Cases 237, 200, and 395 each had, in one eye, corneal or lens opacities precluding visualization of the fundus, with X-ray evidence of intraocular calcium, and retinoma in the other eye. After enucleation for cosmetic purposes, microscopic examination of the phthisical eye in Cases 200 and 295 showed gliosis and calcification; despite the absence of recognizable tumour cells, the pathological appearances are considered to be consistent with necrotic retinoblastoma.

The pedigrees of 3 of the individuals listed in the Table are shown in Fig. 3. In Family I (Case 115, Group C), a mother produced 4 children with retinoblastoma by 2 different fathers; one child died of metastatic retinoblastoma and another probably of pinealoblastoma, though metastatic retinoblastoma could not be ruled out. The mother had no clinical or previous family history of retinoblastoma, but examination of her retinas revealed multiple, bilateral retinomas. In Family II, there are 2 individuals with retinomas in succeeding generations. Case 165 had unilateral retinoblastoma treated by enucleation. When he subsequently produced 2 offspring with retinoblastoma, he was re-examined, and 2 small retinomas were detected in his remaining eye. His daughter (Case 70) also had a unilateral retinoblastoma treated by enucleation. Later examination of her remaining eye, when her own child developed retinoblastoma, revealed 2 retinomas.

### DISCUSSION

We have found 36 cases in the literature of retinal lesions in functional eyes, reported as spontaneous regression of retinoblastoma, which appear to be identical to what we have called "retinomas". Three individuals (Cases 31, 128 and 233) reported in the present study have been previously described (Morris & Lapiana, 1974; Brockhurst & Donaldson, 1970; Rubin & Kaufman, 1969). Although the present study is the largest collection of such cases, it is clear from the literature that retinomas, though rare, have been seen in many centres throughout the world for many years. The excellent clinical descriptions and frequent photographs demonstrate well the characteristics of retinomas.

Twenty-seven of the 36 previously reported cases of retinomas were associated with retinoblastoma: 8 had a family history (Morris & Lapiana, 1974; Brockhurst & Donaldson, 1970; Khodadoust *et al.*, 1977; Smith, 1974; Merin *et al.*, 1965; Hine, 1944), 14 had retinoblastoma in the other eye (Smith, 1974; Fuchs, 1943; Karsgaard, 1971; Meller, 1915; Salzmann, 1921; Seuss & Stutz, 1951; Siegrist, 1912; Von Hippel, 1928; Wüstenberg, 1950) and 5 had both a family history and retinoblastoma in the other eye (Smith, 1974; Purtscher, 1914; Stallard, 1936). The remaining 9 cases of retinoma had no other evidence of retinoblastoma (Rubin & Kaufman, 1969; Smith, 1974; Boniuk & Zimmerman, 1962; Nehen, 1975; Pearce & Gillan, 1972; Sakic, 1959). In the present study, two-thirds of the cases have either immediate family members affected by retinoblastoma, have themselves a diagnosis of retinoblastoma in the other eye, or both. The clearest proof of the association of retinomas and retinoblastoma is Case 115. Since this woman with bilateral retinomas produced 2 children with retinoblastoma by each of 2 husbands, there is little doubt that she carries the gene for the heritable form of retinoblastoma. We conclude from these data that retinomas and retinoblastoma are induced by similar genetic changes. Furthermore, we propose that multiple retinomas, or a single retinoma in the remaining eye of a unilateral retinoblastoma patient, indicate the hereditary form of retinoblastoma. This hypothesis leads to 3 predictions. First, the age at diagnosis for the unilateral retinoblastoma patients in Groups D and E should be more characteristic of bilateral hereditary retinoblastoma than of non-hereditary unilateral disease. The mean age at diagnosis of retinoblastoma in Groups D and E was 1·8 years, a value similar to that reported for bilateral-retinoblastoma patients.

Second, close to 50% of the offspring of individuals in Groups B and D will develop hereditary retinoblastoma. Case 31 represents the first confirmation of this prediction. This person originally was diagnosed with bilateral retinomas in the absence of any family history of retinoblastoma and was placed in Group B. However, this individual recently produced a child who has developed multiple retinoblastoma tumours; Case 31 is now listed in Group C.

Third, on the basis of the observation that only 10% of unilaterally affected patients have the heritable form of retinoblastoma, we predict that only 10% of the individuals with a single retinoma (Group A) may produce children with retinoblastoma. All the children produced by the individuals listed in the Table will be followed to determine the validity of this prediction.

Because of the significance of the diagnosis of retinoma, it is important that the initial identification of a lesion be correct. A retinoma is characteristically a translucent, grey, retinal mass with calcified nodules and an underlying disturbance of pigment epithelium, as shown in Fig. 1. Differential diagnosis in cases without other evidence of retinoblastoma would include larval granuloma without the usual inflammatory reaction, and astrocytic retinal hamartoma. Early astrocytic hamartomas are translucent, but later progress to a denser white appearance with many nodular areas of calcification, classically resembling a mulberry. These may be multiple and associated with systemic signs of tuberous sclerosis. Clinical differentiation of retinoma from active retinoblastoma is also critical. Indeed, retinomas very closely resemble post-irradiation regressed retinoblastoma. Actively growing retinoblastoma has a more opaque, pinkish-white appearance, in contrast to the grey, translucent appearance of retinomas.

The final differentiztion can be made, however, by serial observation. Retinomas do not progress; actively growing tumours will enlarge, spread and affect normal intraocular structures. At the first sign of enlargement, any suspicious lesions must be treated as retinoblastoma. Rychener

(1948) observed malignant retinoblastoma in an eye of a 33-year-old who had been observed for retinoma; it is important that retinomas be observed periodically throughout the patient's lifetime.

The mechanisms by which retinomas arise are not known. The previous term "spontaneous regression of retinoblastoma", implied that some naturally occurring event interfered with proliferation of malignant cells, leading to decreased tumour size. Since it would be unethical to refuse treatment of an obvious tumour, it is difficult to observe regression in retinoblastoma. Wüstenburg (1950) observed 2 individuals at the beginning of World War II with typical retinoblastomas; neither patient could be treated. When seen again 8 years later the lesions in both children had "regressed" and were characteristic of retinomas. These 2 cases represent the only examples, albeit partially documented, of regression of retinoblastoma, without treatment and without phthisis bulbi. Despite the lack of data documenting regression, the conventional wisdom is that retinomas arise by regression of retinoblastoma. If one considers various possible mechanisms for regression, it is difficult to find support for any of them.

Immunological mechanisms are commonly postulated in conversion of retinoblastoma into retinoma. Although Char *et al.* (1974) originally reported specific cytotoxicity in the peripheral blood of patients with retinoblastoma, we have found that peripheral-blood lymphocytes from patients with retinomas had the same cytotoxicity against retinoblastoma as lymphocytes from other retinoblastoma patients and from controls (Gallie *et al.*, 1979*a*). We also observed no correlation of HLA-A or B with retinoblastoma or retinomas (Gallie *et al.*, 1977*b*). At present there is no evidence that immunological mechanisms are involved in retinomas or retinoblastoma.

Verhoeff (1966) suggested that excessive calcification might inhibit growth of retinoblastomas. However, because not all retinomas contain calcium, whereas many retinoblastomas contain large quantities of calcium without any sign of retarded growth, it is unlikely that calcification plays any role in the aetiology of retinomas. Ischaemic necrosis is prominent in most retinoblastomas, and may well explain the "spontaneous regressions" of retinoblastoma which result in phthisis bulbi. However, retinomas have a clinically observable intact blood supply, as demonstrated in Fig. 2, making ischaemia unlikely.

Smith (1974) studied microscopically 2 eyes containing untreated lesions fitting our description of retinomas. He found only small areas of necrosis, and did not detect dividing tumour cells. He characterized the cells as "differentiated neural elements" and proposed that tumour-cell maturation was the mechanism of "regression", drawing a comparison with benign ganglioneuromatous differentiation seen in neuroblastoma. These retinomas, however, did not show the usual pattern of highly differentiated retinoblastoma with Flexner–Wintersteiner rosettes, Homer–Wright rosettes and fleurettes, that has been described by Tso *et al.* (1970) in slow-growing radiation-resistant retinoblastomas. These morphological data, showing that the only retinomas studied histologically contain differentiated elements, suggest that abnormal differentiation may produce benign nodules such as retinomas.

In this context, Knudsen & Meadows (1980) have suggested that benign hyperplasia is often associated with heritable tumours, because the germ-line mutation itself leads to increased proliferation of certain tissue types, depending on the mutation. If any cell in the expanded hyperplastic population acquires a second mutation, they propose that that cell will become malignant. Thus, according to their hypothesis, neuroblastoma IVs, hereditary neurofibromas, C-cell hyperplasia and adrenal medullary hyperplasia are benign hyperplasias resulting from different, single germ-line mutations. Acquisitions of a second mutation in any of these cells will lead to neuroblastoma, neurofibrosarcoma, medullary carcinoma of the thyroid or phaeochromocytoma respectively.

By analogy, one could propose that retinomas are benign hyperplasias resulting from a germ-line mutation affecting retinal differentiation, and that retinoblastomas are the tumours resulting from a second mutation. This model is unlikely for 2 reasons. First, although there is some histological evidence for imperfect differentiation in the non-tumour retina of patients with hereditary retinoblastoma (Uga *et al.*, 1978), most patients have normal visual function and normal electroretinograms in successfully treated eyes. Second, retinomas are rare. Only a small proportion of asymptomatic carriers of the retinoblastoma gene develop retinomas, and when these do arise, few are seen in affected patients. If the germ-line mutation alone was sufficient to induce retinomas, we would expect them to occur frequently and in high numbers in affected patients.

Therefore we propose that retinomas, like retinoblastomas, require another event in addition to the germ-line mutation. This alternative explanation of retinomas is also based on Knudson's 2-mutation hypothesis (Knudson, 1971). Since the frequency of retinoblastoma development drops rapidly after 5 years of age, the target for induction of retinoblastoma must disappear with time; otherwise individuals with retinoblastoma should appear in all age groups, with the incidence gradually increasing with age. The fact that tumours do not appear in adults implies that retinoblasts eventually differentiate into cells in which mutations or other carcinogenic events cannot lead to the development of a tumour. Presumably carcinogenic events occur at random, and may happen at any stage in the differentiation of retinoblasts. If the final mutation occurs in an immature retinoblast, it will lead to retinoblastoma. If the mutation occurs in a partially differentiated cell before terminal differentiation, it could cause abnormal proliferation leading to a hyperplastic nodule of differentiated cells; *i.e.*, a retinoma. If the mutation occurs in a terminally differentated cell, it will have no effect because these cells cannot proliferate.

This explanation fits with the available clinical data on retinomas. In this model both retinoma and retinoblastoma are induced by the same series of mutations, the difference arising from the timing, in terms of embryological retinal development, of the second mutation. Retinoblast cells, capable of giving rise to retinoblastoma, are present for the first few years of life. The low frequency of retinoma compared to retinoblastoma suggests that the transition phase between retinoblast and adult retinal cell may be short.

By this model it is entirely possible for retinomas and retinoblastoma to co-exist in the same patient, though if present in the same eye the retinoblastoma would overshadow the retinoma, making its diagnosis unlikely. The nonspecific, non-photoreceptor type of "differentiated neurons" seen in the retinomas examined histologically by Smith (1974) would be consistent with benign proliferation of an early retinal cell.

This research was supported in part by Fight-for-Sight, Inc., New York, Grant No. 6572 and by the Ontario Cancer Treatment and Research Foundation, Grant No. 399. Lee Buckhough, Harkness Eye Institute, played an invaluable role in collecting much of the data on patients reported in this paper.

### REFERENCES

ANDERSEN, S. R. Y. & JENSEN, Q. A. (1974) Retinoblastoma with necrosis of central retinal artery and vein and partial spontaneous regression. *Acta Ophthalmol.*, **52**, 182.

BONIUK, M. & ZIMMERMAN, L. E. (1962) Spontaneous regression of retinoblastoma. *Int. Ophthalmol. Clin.*, **2**, 525.

BROCKHURST, R. J. & DONALDSON, D. (1970) Spontaneous resolution of probable retinoblastoma. *Arch. Ophthalmol.*, **84**, 388.

CHAR, D. H., ELLSWORTH, R., RABSON, A. S., ALBERT, D. M. & HERBERMAN, R. B. (1974) Cell mediated immunity to a retinoblastoma tissue culture line in patients with retinoblastoma. *Am. J. Ophthalmol.*, **78**, 5.

COLE, W. H. (1974) Spontaneous regression of cancer; Metabolic triumph of the host? *Ann. N.Y. Acad. Sci.*, **230**, 111.

Fuchs, A. (1943) *Die Erkrankungen des augen Hintergrundes.* Vienna: Deuticke. p. 172.

Gallie, B. L., Dupont, B., Whitsett, C., Kitchen, F. D., Ellsworth, R. M. & Good, R. A. (1977*a*) Histocompatibility typing in spontaneous regression of retinoblastoma. In *HLA and Malignancy.* New York: Alan R. Liss. p. 229.

Gallie, B., Wong, J. J. Y., Ellsworth, R. M., Dupont, B. & Good, R. A. (1977*b*) Immunological mechanisms in spontaneous regression of retinoblastoma. In *Immunology and Immunopathology of the Eye,* (Eds Silverstein and O'Connor). New York: Masson. p. 190.

Hine, M. L. (1944) A sequel to the spontaneous cure of "glioma of the retina" reported in *Transactions,* 1937. *Trans. Ophthalmol. Soc. U.K.,* **64,** 99.

Karsgaard, A. A. (1971) Spontaneous regression of retinoblastoma. *Can. J. Ophthalmol.,* **6,** 218.

Khodadoust, A. A., Roozitalab, H. M., Smith, R. E. & Green, W. R. (1977) Spontaneous regression of retinoblastoma. *Surv. Ophthalmol.,* **21,** 467.

Knudson, A. G. (1971) Mutation and cancer: Statistical study of retinoblastoma. *Proc. Natl Acad. Sci.,* **68,** 820.

Knudson, A. G. & Meadows, A. T. (1980) Regression of neuroblastoma IV-S: A genetic hypothesis. *N. Engl. J. Med.,* **302,** 1254.

Meller, J. (1915) On the retrogression of retinal glioma. *Am. J. Ophthalmol.,* **32,** 193.

Merin, S., Robinson, E. & Landau, J. (1965) Spontaneous healed retinoblastoma as a factor in dominant heredity. *J. Pediatric. Ophthalmol.,* **2,** 43.

Morris, W. E. & Lapiana, F. G. (1974) Spontaneous regression of bilateral multifocal retinoblastoma with preservation of normal visual acuity. *Ann. Ophthalmol.,* **6,** 1192.

Nehen, J. H. (1975) Spontaneous regression of retinoblastoma. *Acta Ophthalmol.,* **53,** 637.

Pearce, W. G. & Gillan, J. G. (1972) Bilateral spontaneous regression of retinoblastoma. *Can. J. Ophthalmol.,* **7,** 234.

Purtscher, O. (1914) Zur Kenntnis des Markesschwamms der Netzhaut und seiner spontaner Ruckbildung. *Zentralbl. Prakt. Augenheilk,* **38,** 193.

Rubin, M. L. & Kaufman, H. E. (1969) Case report of spontaneously regressed retinoblastoma. *Arch. Ophthalmol.,* **81,** 442.

Rychener, R. O. (1948) Retinoblastoma in the adult. *Trans. Am. Ophthalmol. Soc.,* **46,** 318.

Sakic, N. (1959) Ein Fall von ruckgebildten doppelzeitigen Glioma retinae. *Zentralbl. Ophthalmol.,* **78,** 45.

Salzmann, E. (1921) Neubildungen der Netzhaut. *Lehrbuch der Augenheilk,* **13,** 589.

Seuss, A. & Stutz, E. (1951) Zur Strahlenbehandlung des Netzhautglioms. *Strahlentherapie,* **85,** 589.

Siegrist, A. (1912) Demonstration verschiedener ophthalmoskopischer Befunde, microskopischer Preparate und eines neuen Tasterzirkels zu orbital Messungen. *Beitr. Ophthalmol.,* **33,** 353.

Smith, J. L. S. (1974) Histology and spontaneous regression of retinoblastoma. *Trans. Ophthalmol. Soc. U.K.,* **94,** 953.

Stallard, H. B. (1936) Glioma retinae: Spontaneous cure during scarlet fever. *Proc. R. Soc. Med., Sect. Ophthalmol.,* **14,** 11.

Tso, M. O. M., Zimmerman, L. E., Fine, B. S. & Ellsworth, R. M. (1970) A cause of radioresistance in retinoblastoma: Photoreceptor differentiation. *Trans. Am. Acad. Ophthalmol. Otolaryngol.,* **73,** 959.

Uga, S., Fujiwara, N., Ishikawa, S. & Shimizu, K. (1978) Electron microscopy of the retina with retinoblastoma. *Jap. J. Ophthalmol.,* **22,** 382.

Verhoeff, F. H. (1966) Retinoblastoma undergoing spontaneous regression. *Am. J. Ophthalmol.,* **62,** 573.

Von Hippel, E. (1928) Selbtheilung eines Netzhautgliome. *Klin. Monatsbl. Augenheilk.,* **81,** 30.

Wüstenberg, W. (1950) Uber zwei Falle von spontan geheiltem Netzhautgliom. *Klin. Monatsbl. Augenheilk.,* **117,** 423.


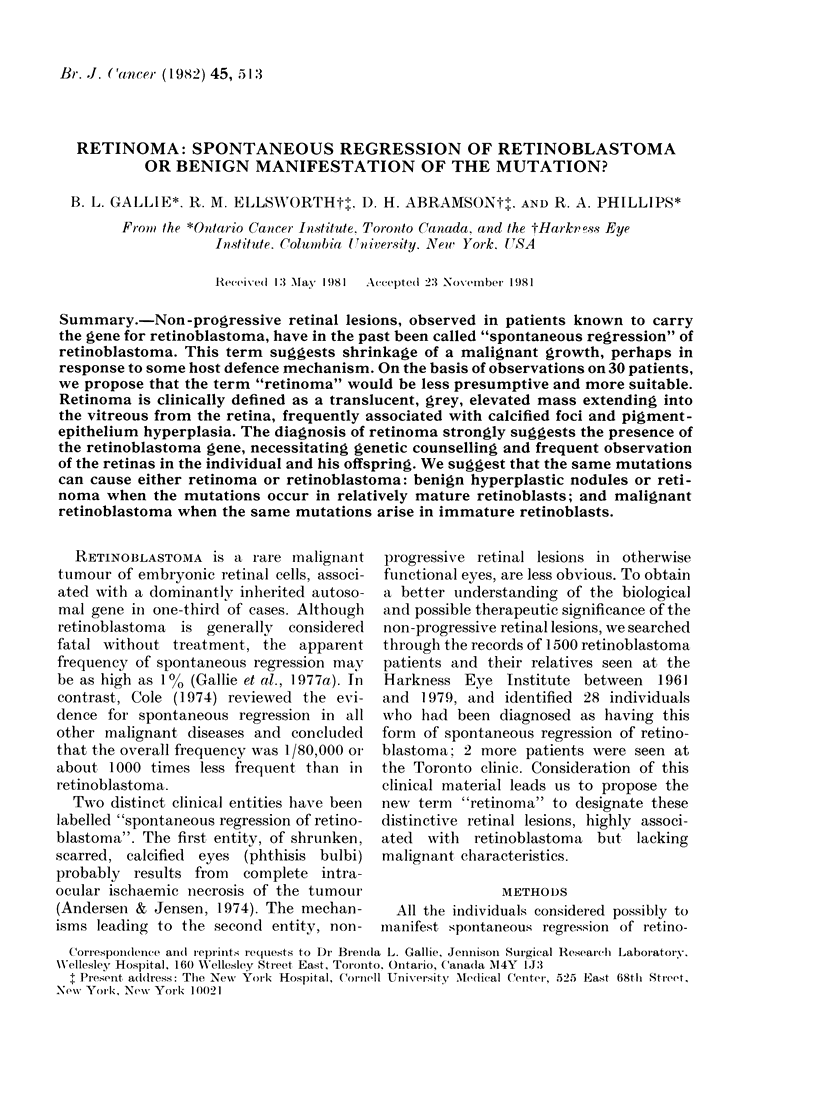

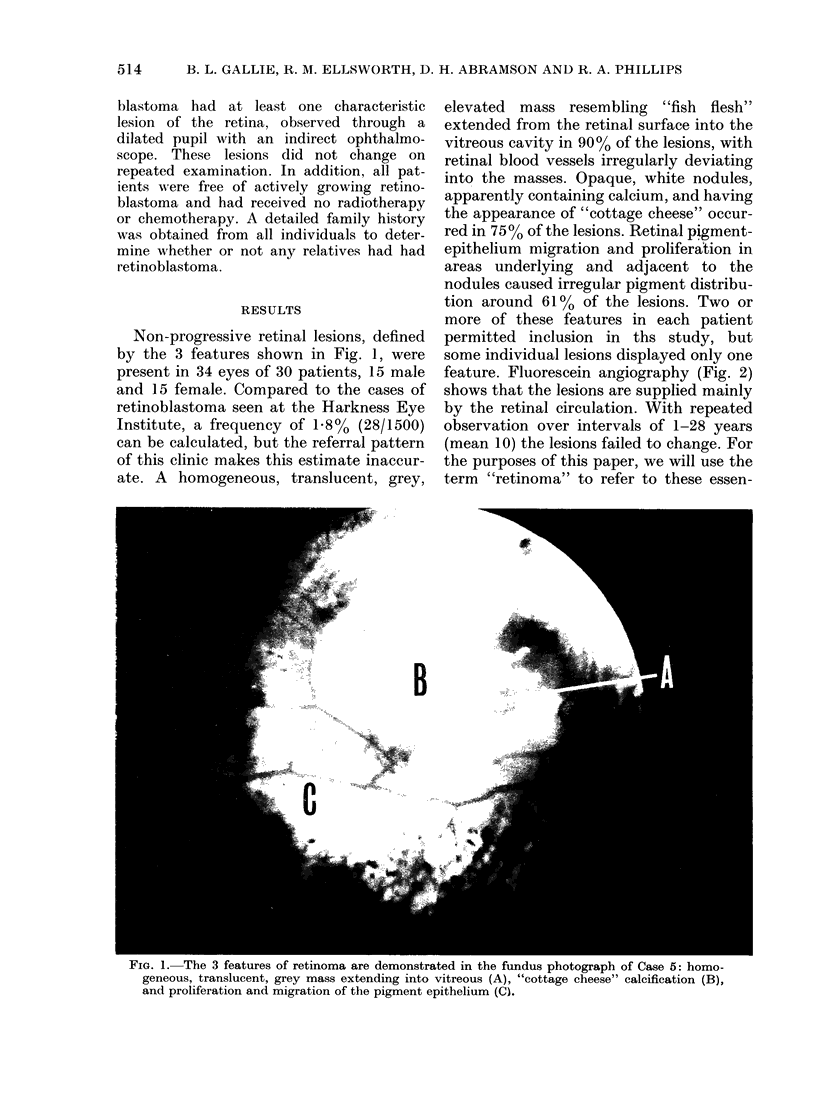

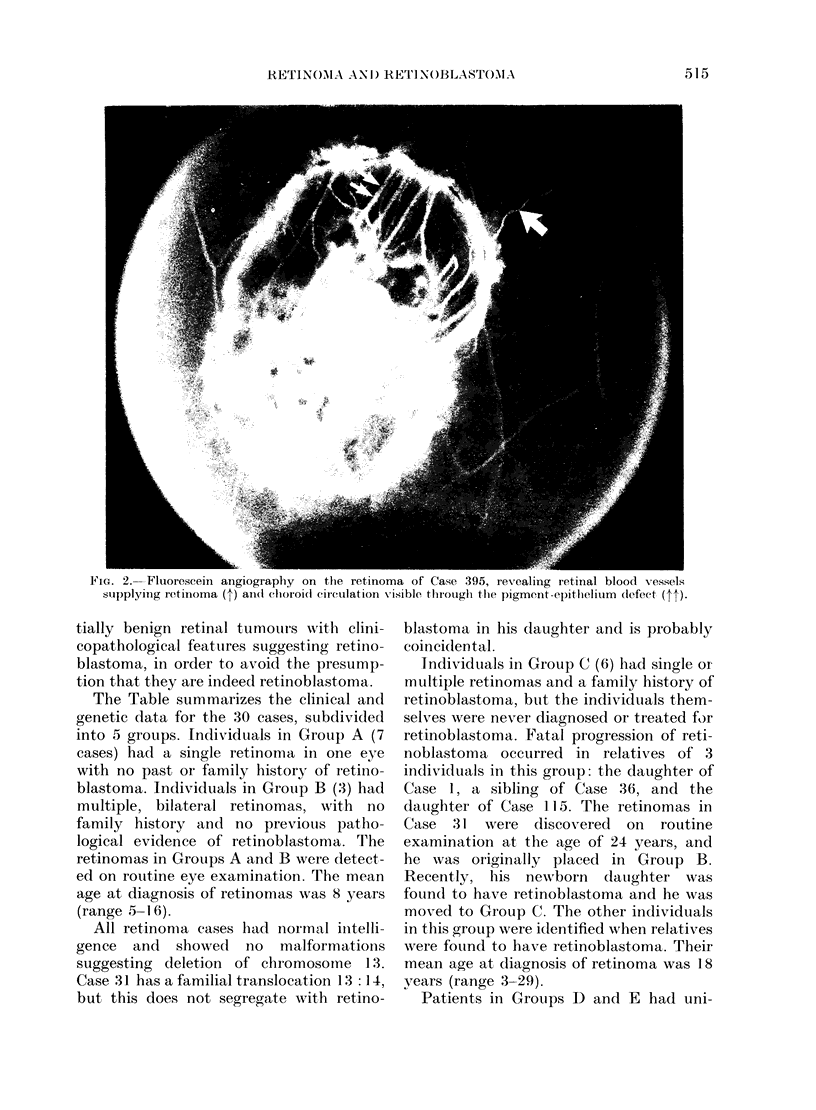

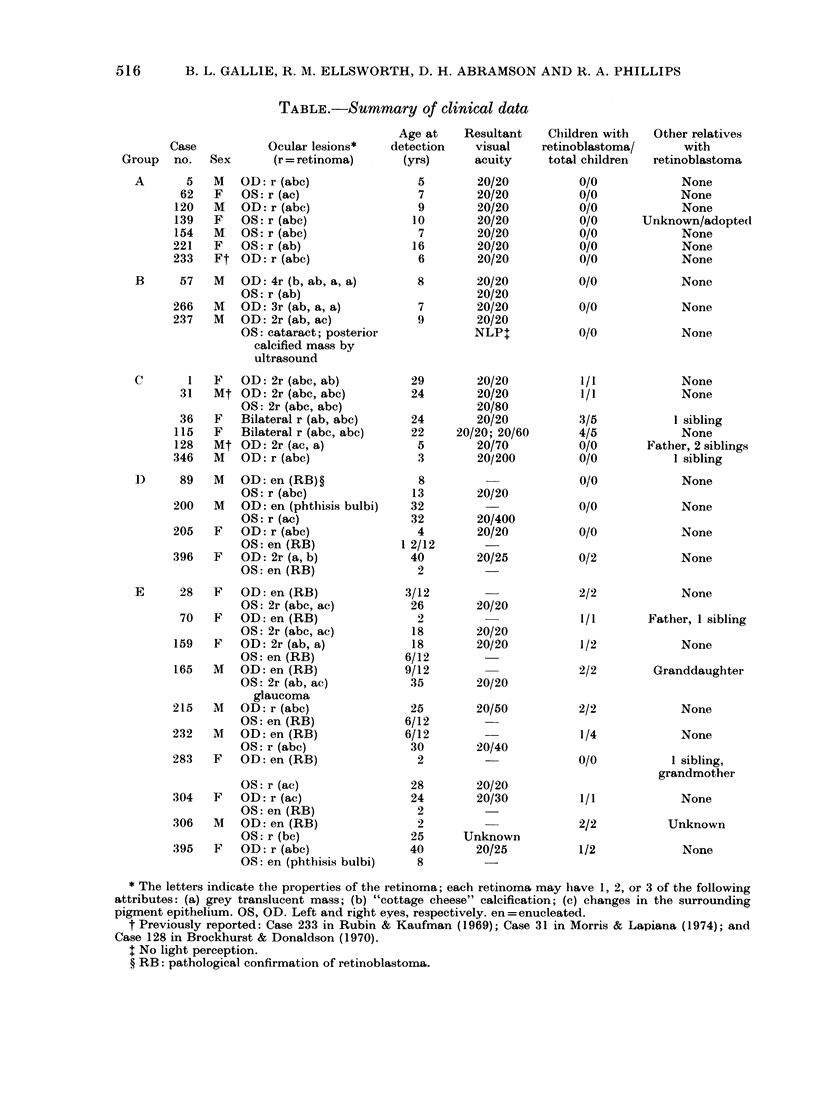

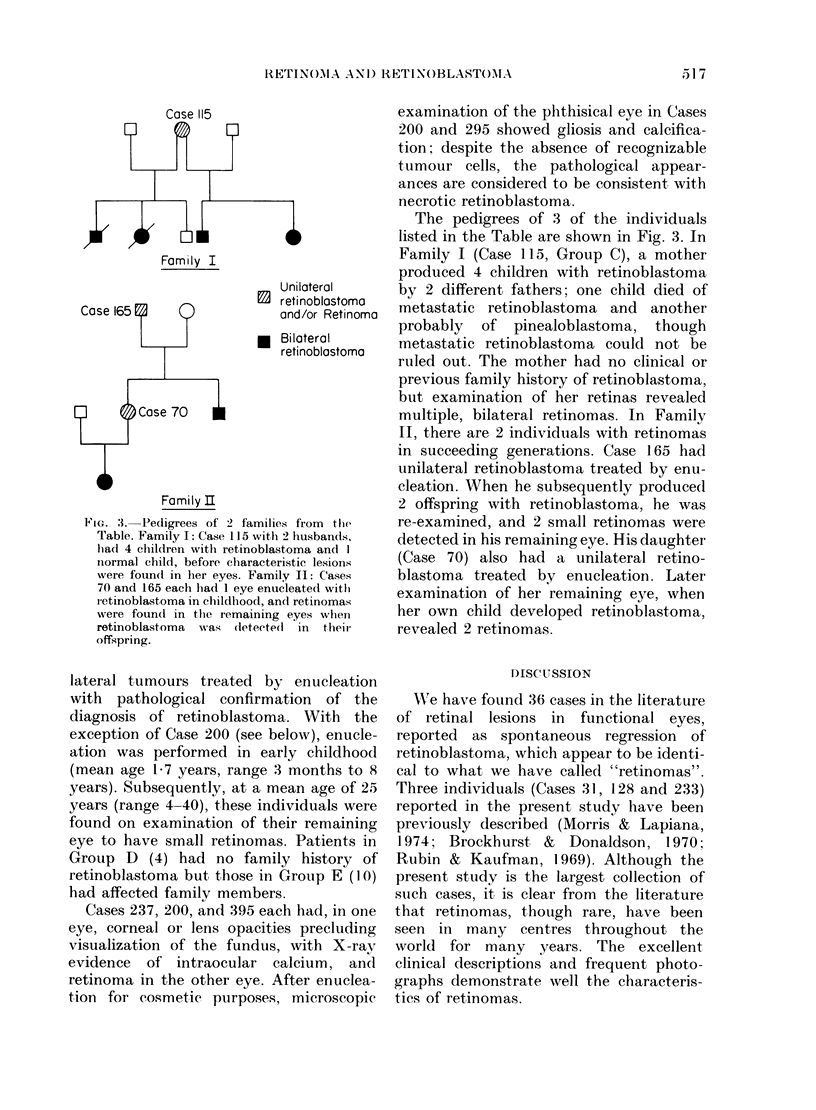

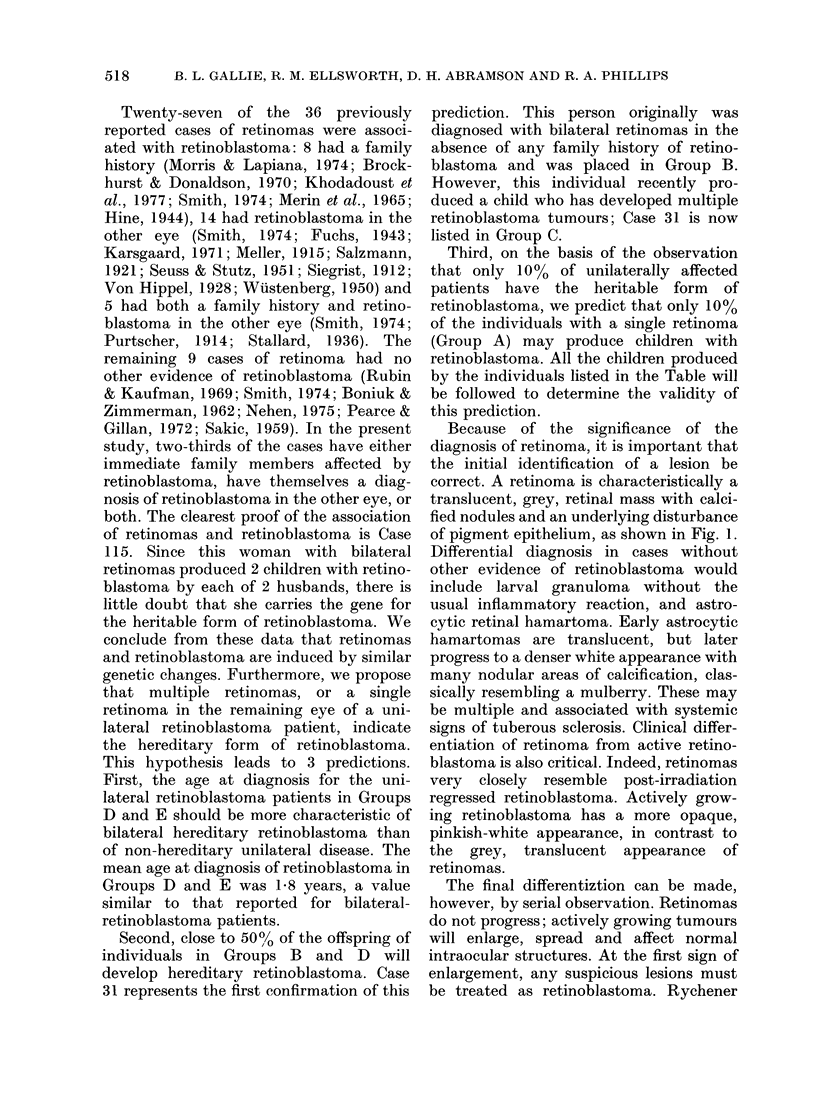

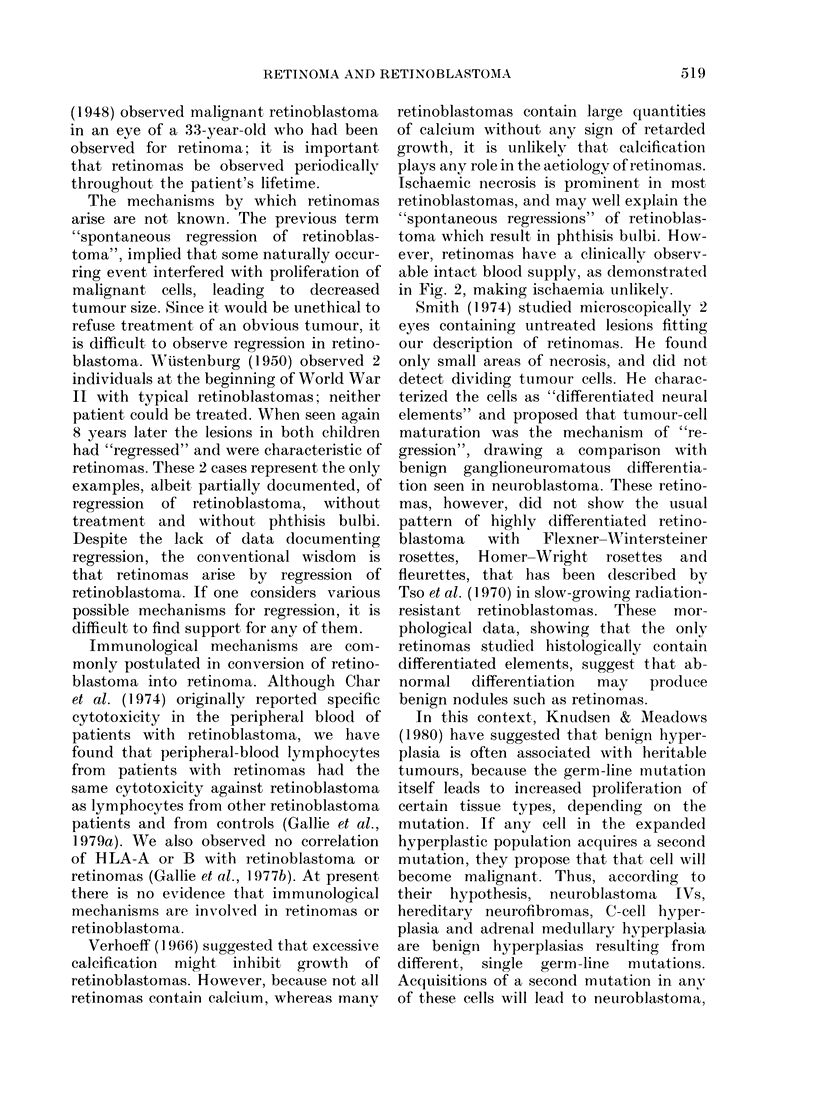

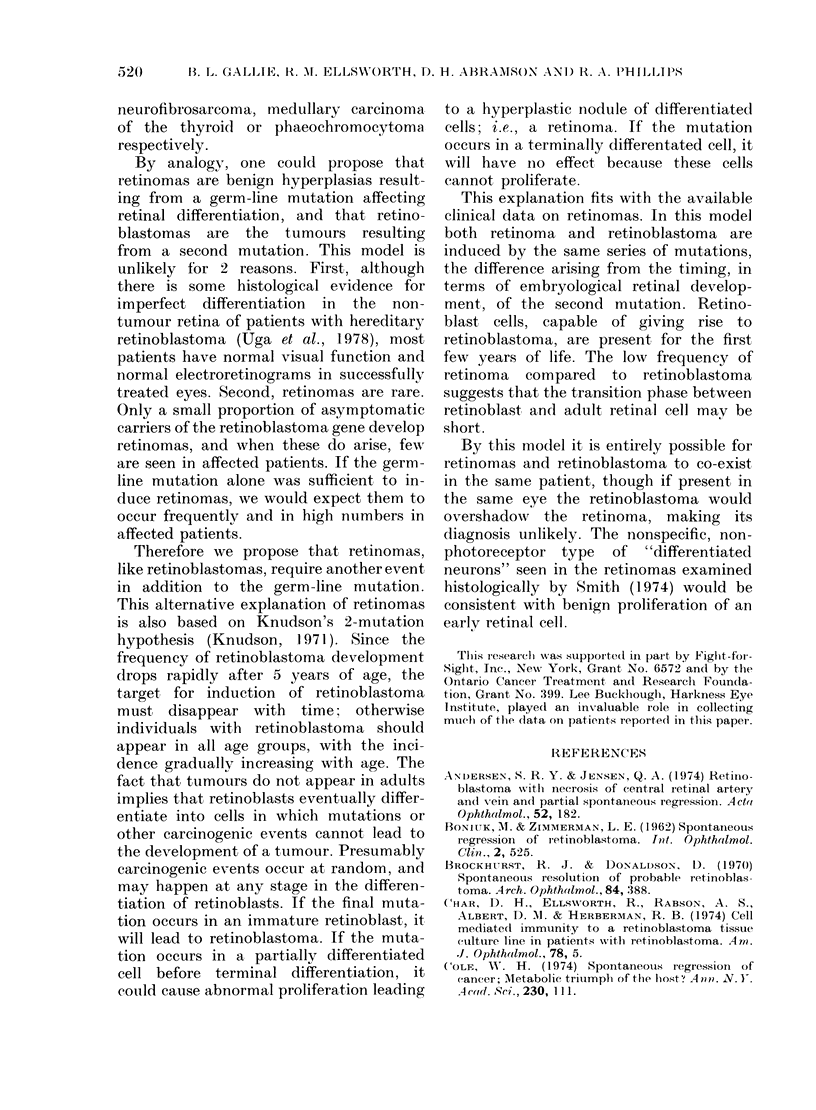

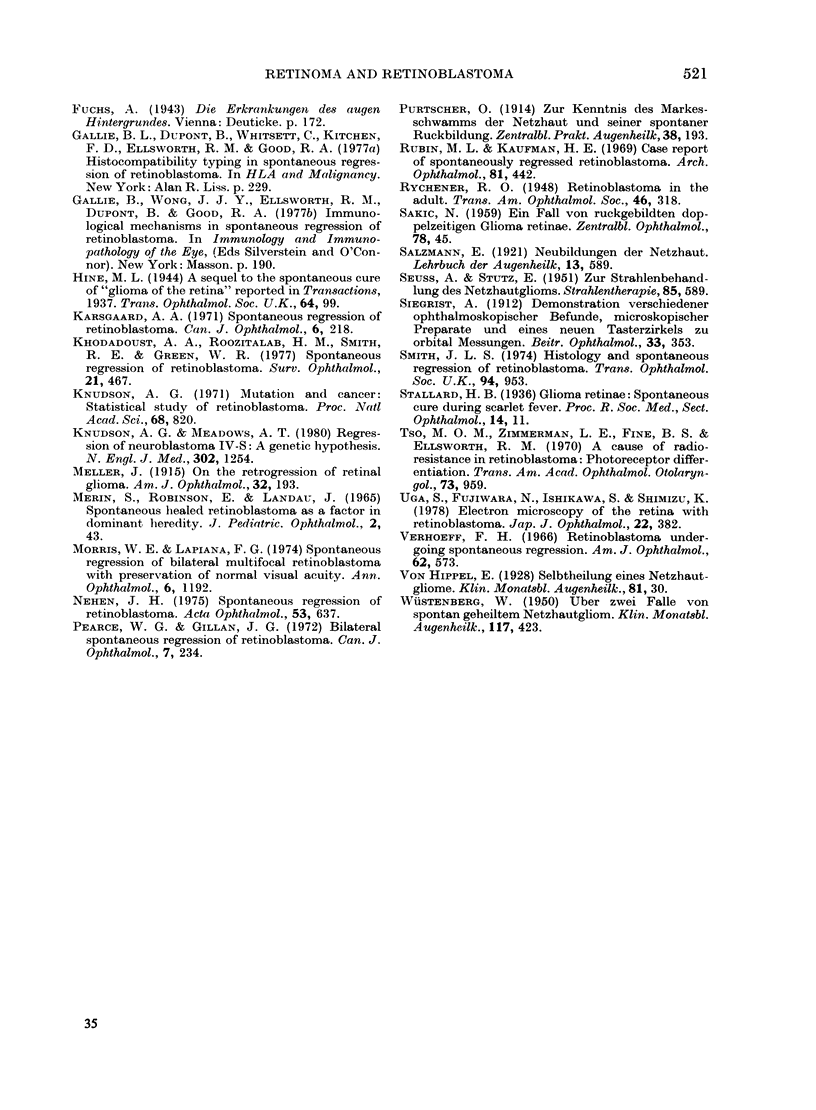


## References

[OCR_01026] Karsgaard A. T. (1971). Spontaneous regression of retinoblastoma. A report of two cases.. Can J Ophthalmol.

[OCR_01030] Khodadoust A. A., Roozitalab H. M., Smith R. E., Green W. R. (1977). Spontaneous regression of retinoblastoma.. Surv Ophthalmol.

[OCR_01041] Knudson A. G., Meadows A. T. (1980). Sounding board. Regression of neuroblastoma IV-S: a genetic hypothesis.. N Engl J Med.

[OCR_01036] Knudson A. G. (1971). Mutation and cancer: statistical study of retinoblastoma.. Proc Natl Acad Sci U S A.

[OCR_01102] Lindley J., Smith S. (1974). Histology and spontaneous regression of retinoblastoma.. Trans Ophthalmol Soc U K.

[OCR_01056] Morris W. E., LaPiana F. G. (1974). Spontaneous regression of bilateral multifocal retinoblastoma with preservation of normal visual acuity.. Ann Ophthalmol.

[OCR_01066] Pearce W. G., Gillan J. G. (1972). Bilateral spontaneous regression of retinoblastoma.. Can J Ophthalmol.

[OCR_01075] Rubin M. L., Kaufman H. E. (1969). Spontaneously regressed probable retinoblastoma. Report of a case.. Arch Ophthalmol.

[OCR_01080] Rychener R. O. (1948). Retinoblastoma in the Adult.. Trans Am Ophthalmol Soc.

[OCR_01093] SEUSS A., STUZ E. (1951). Zur Strahlenbehandlung des Netzhautglioms.. Strahlentherapie.

[OCR_01112] Ts'o M. O., Zimmerman L. E., Fine B. S., Ellsworth R. M. (1970). A cause of radioresistance in retinoblastoma: photoreceptor differentiation.. Trans Am Acad Ophthalmol Otolaryngol.

[OCR_01124] Verhoeff F. H. (1966). Retinoblastoma undergoing spontaneous regression. Calcifying agent suggested in treatment of retinoblastoma.. Am J Ophthalmol.

[OCR_01133] WUSTENBERG W. (1950). Uber zwei Fälle von spontan geheiltem Netzhautgliom.. Klin Monbl Augenheilkd Augenarztl Fortbild.

